# Validation of a modern second‐check dosimetry system using a novel verification phantom

**DOI:** 10.1002/acm2.12025

**Published:** 2017-01-19

**Authors:** Daniel G. McDonald, Dustin J. Jacqmin, Christopher J. Mart, Nicholas C. Koch, Jean L. Peng, Michael S. Ashenafi, Mario A. Fugal, Kenneth N. Vanek

**Affiliations:** ^1^ Department of Radiation Oncology Medical University of South Carolina Charleston SC USA

**Keywords:** eclipse acuros, IMRT, Mobius, Mobius verification phantom, VMAT

## Abstract

**Purpose:**

To evaluate the Mobius second‐check dosimetry system by comparing it to ionization‐chamber dose measurements collected in the recently released Mobius Verification Phantom™ (MVP). For reference, a comparison of these measurements to dose calculated in the primary treatment planning system (TPS), Varian Eclipse with the AcurosXB dose algorithm, is also provided. Finally, patient dose calculated in Mobius is compared directly to Eclipse to demonstrate typical expected results during clinical use of the Mobius system.

**Methods:**

Seventeen anonymized intensity‐modulated clinical treatment plans were selected for analysis. Dose was recalculated on the MVP in both Eclipse and Mobius. These calculated doses were compared to doses measured using an A1SL ionization‐chamber in the MVP. Dose was measured and analyzed at two different chamber positions for each treatment plan. Mobius calculated dose was then compared directly to Eclipse using the following metrics; target mean dose, target D95%, global 3D gamma pass rate, and target gamma pass rate. Finally, these same metrics were used to analyze the first 36 intensity modulated cases, following clinical implementation of the Mobius system.

**Results:**

The average difference between Mobius and measurement was 0.3 ± 1.3%. Differences ranged from −3.3 to + 2.2%. The average difference between Eclipse and measurement was −1.2 ± 0.7%. Eclipse vs. measurement differences ranged from −3.0 to −0.1%. For the 17 anonymized pre‐clinical cases, the average target mean dose difference between Mobius and Eclipse was 1.0 ± 1.1%. Average target D95% difference was ‐0.9 ± 2.0%. Average global gamma pass rate, using a criteria of 3%, 2 mm, was 94.4 ± 3.3%, and average gamma pass rate for the target volume only was 80.2 ± 12.3%. Results of the first 36 intensity‐modulated cases, post‐clinical implementation of Mobius, were similar to those seen for the 17 pre‐clinical test cases.

**Conclusion:**

Mobius correctly calculated dose for each tested intensity modulated treatment plan, agreeing with measurement to within 3.5% for all cases analyzed. The dose calculation accuracy and independence of the Mobius system is sufficient to provide a rigorous second‐check of a modern TPS.

## 1 Introduction

Mobius3D and MobiusFX are two components of the Mobius second‐check dosimetry system produced by Mobius Medical Systems (Mobius Medical Systems, Houston, TX, USA). Mobius3D performs a full recalculation of dose on the patient CT‐dataset using treatment parameters exported from the primary treatment planning system (TPS) and an independent convolution‐superposition algorithm.^1^ MobiusFX allows for quality assurance of the treatment plan by offering a “delivered dose” calculation, generated using the Mobius3D model and delivery log files produced by the treatment machine. In addition, the MobiusFX module provides automatic “delivered dose” calculation on the Mobius Verification Phantom (MVP). The MVP is a Virtual Water™ dosimetry phantom with seven pre‐determined ionization chamber positions for absolute dose measurement. The expected dose at each of these chamber positions is automatically calculated in the MobiusFX module, using the Mobius3D beam model and treatment delivery log files. For the purposes of this work, both the MobiusFX and Mobius3D modules will be referred to as “Mobius”.

In order for a second‐check dosimetry system to provide useful information to the user, the user must have confidence in the ability of the second‐check system to accurately calculate dose. While second‐check systems are not expected to approach the dosimetric accuracy of the primary TPS, their accuracy must be such that the number of “false positives” produced does not obscure any true problems with the primary TPS. Ideally, a treatment plan failing a second‐check dosimetry calculation should be an unusual occurrence, prompting a thorough investigation. If the second‐check system lacks the ability to accurately calculate dose for complex modern treatment plans, failures will be routine and indeterminate, rendering the second‐check largely meaningless.^2^

Excellent work has previously been published examining the accuracy of Mobius as compared to various clinical treatment planning systems for intensity modulated treatment plans. Francisco Clemete‐Gutierrez and Consuelo Perez‐Vara compared Mobius to the Monaco TPS and dosimetric measurements taken using the IBA EasyCube Phantom (IBA Dosimetry, Schwarzenbruck, Germany).^3^ Works by Nelson et al. and Jonas D. Fontenot compare Mobius to the Pinnacle TPS (Philips International, Amsterdam, The Netherlands).^4^, ^5^, ^6^ Dosimetric measurements for these works were taken using various solid water phantoms. Kisling et al. presented work comparing Mobius to the Pinnacle TPS using multiple phantoms from the Radiological Physics Center equipped with TLDs.^7^ Other works have evaluated the beam model in Mobius by directly comparing dose calculated in Mobius to dose calculated in the Varian Eclipse TPS (Varian Medical Systems, Las Vegas, NV, USA).^8^, ^9^

In this work, ionization‐chamber‐based absolute dose measurements of previously treated, clinical intensity‐modulated treatment plans, taken in the recently released MVP, were used to validate the accuracy of the Mobius (v1.5.3) second‐check dosimetry system. In addition, these measurements were compared to our primary TPS, Eclipse, utilizing the AcurosXB (v.11) dose calculation engine. We believe this work is the first to be published evaluating the accuracy of dose calculated by the Mobius second check system compared to that calculated by the modern AcurosXB dose engine, using dose measured in the commercially available MVP as a standard. Finally, dose calculated in Mobius was compared to dose calculated in Eclipse for these plans. Following this pre‐clinical validation, we implemented the Mobius second‐check dosimetry system clinically. Data are presented on the agreement between Mobius and Eclipse for our first 36 clinical cases.

For this study, treatment log files were used solely to facilitate calculation of dose on the MVP within the MobiusFX module. Mobius utilizes the delivery information, reported in the log‐file, when calculating dose within the MobiusFX module. There is an ongoing discussion within the medical physics community regarding the validity of log‐file analysis for patient‐specific quality assurance (QA) of modulated treatment beams.^10^, ^11^, ^12^ Publications examining the reliability and effectiveness of this QA method are available for review.^13^, ^14^, ^15^, ^16^, ^17^, ^18^, ^19^, ^20^

## 2 Methods

### 2.A Mobius commissioning

The Mobius beam model is created primarily using accelerator‐specific universal beam data, provided by Mobius. While this model can be customized to better fit the user's individual treatment machine, Mobius Medical Systems recommends minimizing the amount of customization performed by the end‐user, to preserve the independence of Mobius as a second‐check system. We chose not to customize our beam model for this reason. Mobius also allows for the addition of a dosimetric leaf gap (DLG) correction value for each beam model. This DLG correction should not be viewed in the same way as the DLG parameter in the Eclipse treatment planning system. In Eclipse, the DLG is a multi‐leaf collimator (MLC) positional offset designed to account for additional beam transmission through MLCs with rounded leaf tips.^21^ In Mobius, the DLG correction factor is an *additional* factor added to an internal DLG already present in the Mobius system. The internal DLG is not visible to the user. Mobius Medical Systems recommends using ionization chamber‐based dose measurements to determine the optimal DLG correction factor. Prior to beginning this study, the DLG correction factor was optimized for each Mobius beam model, following the vendor's recommended procedure. The results reported here were calculated using optimum DLG correction values of 1.1 and −0.7 mm for 6 and 10 MV, respectively. Readers attempting to replicate our results should first determine the optimal DLG correction value for their specific treatment machine.

### 2.B Plan selection

For pre‐clinical validation, 17 anonymized intensity‐modulated treatment plans were selected for analysis. All plans were generated in Eclipse. Dose was calculated with the AcurosXB algorithm (v11). The treatment machine for all plans was a Varian TrueBeam accelerator with high‐definition (0.25 and 0.5 cm) MLCs. The accelerator is equipped with 6 and 10 MV flattened photon beams. Plans were chosen to encompass a clinically relevant variety. Nine plans used volumetric modulated arcs (VMAT) for delivery. Of the VMAT plans, six used a beam energy of 6 MV, and three used a beam energy of 10 MV. The eight remaining plans utilized sliding window intensity‐modulation (IMRT) at static gantry angles for delivery. Of the IMRT plans, two used 6 MV, three used 10 MV, and three were mixed‐beam (6 and 10 MV). Both IMRT and VMAT plans incorporated tracking of the primary jaws to minimize MLC leakage. Treatment plan characteristics for these 17 cases can be found in Table 1.

**Table 1:** Characteristics for plans analyzed during pre‐clinical validation**.**

Plan number	Treatment modality	Treatment site	Beam energy (MV)	Number of fields/arcs
1	VMAT	Head and neck	6	2
2			6	2
3		Brain	6	4
4			6	3
5		Prostate	10	2
6			10	2
7		Lung	10	2
8			6	2
9		Mediastinum	6	2
10	IMRT	Lung	6	5
11			6	5
12			10	5
13			10	5
14			10	6
15			6/10	4/2
16			6/10	3/3
17			6/10	3/3

For the post‐implementation clinical plan comparison, the first 36 treatment plans evaluated using Mobius were chosen for inclusion in this study. The only requirement for inclusion was intensity modulation; this included IMRT as well as VMAT. Of these 36 clinical cases, 33 used a beam energy of 6 MV, one used a beam energy of 10 MV, and two used mixed energy (6 and 10 MV).

### 2.C Pre‐clinical validation: determination of phantom dose in eclipse

**Figure 1:**
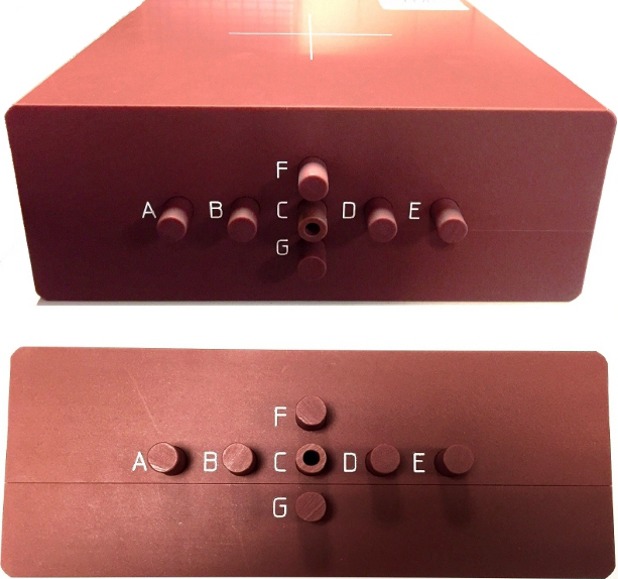
Mobius MVP. Mobius MVP with seven available ionization chamber positions.

For each of the 17 selected treatment plans, a verification plan was generated in the Eclipse treatment planning system. Verification plans were created by copying the beams or arcs onto a CT‐dataset of the MVP™ while maintaining the original MLC segments and beam monitor units. As described previously, the MVP is a Virtual Water™ phantom with seven available positions for an ionization chamber, labeled A through G, as well as a space for film insertion if needed. Our MVP has been drilled for use with an ADCL‐calibrated Standard Imaging A1SL ionization chamber (Standard Imaging, Madison, WI, USA). The MVP is shown in Fig. 1. Dose was calculated on the MVP for each verification plan utilizing the AcurosXB algorithm. Volume‐averaged doses at two selected chamber positions were determined by contouring volumes equal to the active volume of the A1SL chamber in the appropriate locations. Chamber positions were chosen within the treatment area, avoiding regions with steep dose gradients.

### 2.D Pre‐clinical validation: measurement of dose in phantom

The MVP, equipped with an ADCL‐calibrated Standard Imaging A1SL ionization‐chamber, was positioned on the TrueBeam accelerator. The A1SL ionization chamber is constructed of air‐equivalent plastic, and has a collection volume of 0.053 cc, with a collection diameter and length of 1.0 and 4.4 mm, respectively. Prior to delivery of the planned treatment fields, an open 10 × 10 cm field was measured with the chamber at the central position (position C). Expected dose to chamber position C for this beam geometry was calculated by hand using machine commissioning data. This expected dose and the charge collected were used to determine a dose to charge ratio. This process was performed for both the 6 and 10 MV beams.

Intensity modulated plans were then delivered. Measured charge and the previously calculated dose to charge ratios were used to determine measured dose. Each plan was delivered twice, so that dose could be measured for each plan at each of the two selected chamber positions. The 6 and 10 MV portions of mixed‐beam IMRT plans were measured separately, then summed to determine total dose.

### 2.E Pre‐clinical validation: determination of phantom dose in Mobius

The seventeen selected treatment plans were individually exported from Eclipse to the Mobius second‐check system (v1.5.3). Upon receiving the necessary DICOM information (CT‐dataset, RT plan, structure set, and dose), Mobius automatically began a secondary dose calculation on the patient CT using its independent convolution‐superposition algorithm. Treatment log files, generated by the TrueBeam accelerator while measuring dose on the MVP, were also imported into Mobius. Equipped with these log files, Mobius performed an additional dose calculation on an internal model of the MVP Phantom, determining dose at each of the seven available ionization chamber positions.

### 2.F Pre‐clinical validation: MVP dose comparison

For each treatment plan, doses at each of the two selected chamber positions in the MVP, as determined by Eclipse and Mobius, were compared to the doses measured with the A1SL ionization chamber. A percent difference was determined for each dose comparison.

### 2.G Pre‐clinical validation: Mobius vs. AcurosXB test case analysis

For each of the 17 validation treatment plans, the dose distributions calculated on the patient's CT‐dataset by Mobius and AcurosXB were compared using the following metrics: Target mean dose percent difference, target D95% percent difference, global 3D gamma pass rate over the entire dataset, and gamma pass rate over the target. A gamma criterion of 3%, 2 mm was used for this pre‐clinical gamma analysis. For patients with multiple targets, the greatest disagreement was reported.

### 2.H Post‐implementation: Mobius vs. AcurosXB clinical use analysis

Following pre‐clinical validation, we began routine use of the Mobius second check system. To provide the reader with typical outcomes that can be expected upon clinical implementation, results for the first 36 intensity modulated clinical cases were analyzed. The dose distributions calculated on each patient's CT‐dataset by Mobius and AcurosXB were compared using the following metrics: Target mean dose percent difference, target D95% percent difference, global 3D gamma pass rate over the entire dataset, gamma pass rate over the target, and point‐dose difference at a selected calculation point. A gamma criterion of 5%, 2 mm was used. This increased gamma tolerance, along with a point‐dose comparison were added to better correlate the Mobius results with our previous second check system. For patients with multiple targets, the greatest disagreement was recorded. The average, maximum, minimum, and standard deviation were calculated for each metric.

## 3 Results and discussion

### 3.A Pre‐clinical validation: MVP dose comparison

**Figure 2:**
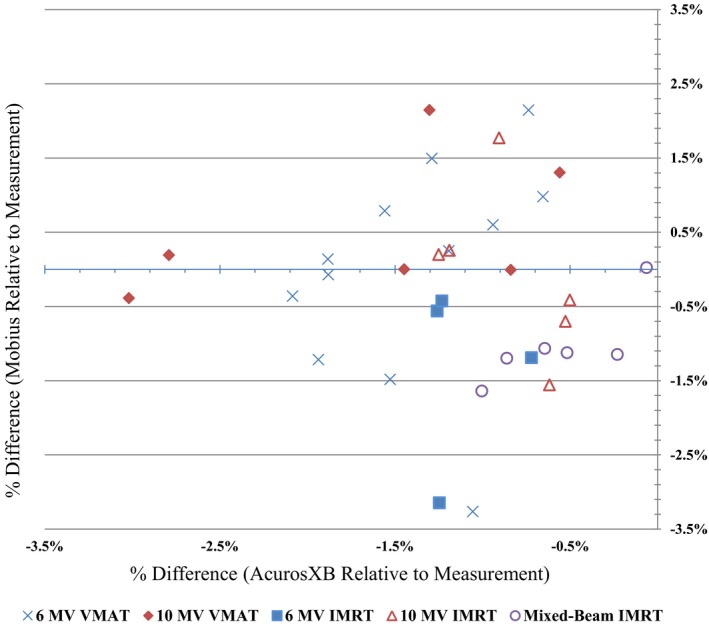
Eclipse AcurosXB and Mobius calculated dose compared to measured dose. Percent differences between dose calculated with Eclipse AcurosXB and Mobius, and dose measured with ionization chamber in the MVP. Eclipse vs. measurement percent difference is plotted vertically on the *Y* axis, while Mobius vs. measurement is plotted horizontally on the *X* axis. Data points are categorized by treatment planning technique.

Percent differences relative to measurement for both AcurosXB and Mobius for each measured point can be found in Fig. 2. Differences are categorized by treatment technique and beam energy. AcurosXB consistently underestimated dose compared to measurement, with differences ranging from −0.1 to −3.0%. The average difference between AcurosXB and measurement was −1.2 ± 0.7%. Mobius both over and underestimated dose compared to measurement. Differences ranged from −3.3 to + 2.1%. The average difference between Mobius and measurement was −0.3 ± 1.3%. These results are similar to those seen by others who have compared Mobius to ionization chamber measurements. Table 2 provides a comparison of the results seen in this work and other similar studies.

**Table 2:** Summary of selected publications evaluating Mobius vs. ionization‐chamber measurement

Reference	Plan type	Delivery technique	Number of plans analyzed	Points analyzed per plan	Ionization chamber	Phantom	Reported percent difference range (%)	Reported average percent difference ± standard deviation (%)
Nelson et al. 4	Clinical	VMAT	12	1	IBA CC04	Rectangular solid water	NA	+1.5 ± 1.0^a^
		IMRT	28					−0.2 ± 1.0^a^
Fontenot et al. 5	TG 119	VMAT	4	2–3	Standard Imaging A1SL	Cylindrical solid water	−4.2 to +2.3	−1.6 ± 2.3^b^
		IMRT	4				−3.5 to +5.5	−0.6 ± 2.8^b^
Clemente‐Gutierrez et al. 3	TG 119	VMAT	4	1	IBA CC04	IBA easycube™	−1.0 to +2.8^c^	+0.9 ± 1.7^b^^,^ c
	Clinical		12				−1.7 to +2.0^c^	+0.1 ± 1.0^b^^,^ c
Present study	Clinical	VMAT	9	2	Standard Imaging A1SL	Mobius MVP™	−3.3 to +2.1	+0.2 ± 1.3
		IMRT	8				−3.1 to +1.8	−0.7 ± 1.0

a Nelson et al. reported only average differences.

b Fontenot et al. and Clemente‐Gutierrez et al. did not report average percent differences. Average percent differences reported here were calculated from available data.

c Clemente‐Gutierrez et al. reported differences in scatter‐plot form, so approximate results are shown here.

Overall, we found AcurosXB to be consistently low compared to measurement with the MVP, while Mobius dose differences were more varied. This is likely due to tuning of the DLG correction factor in Mobius. This correction factor was adjusted based on ionization chamber measurements, similar to those undertaken in this study, in an effort to create an even distribution of dose differences as calculated by Mobius. The success of this tuning can be seen in the results presented here: the average difference from Mobius to ion chamber measurement is near zero. However, the standard deviation of the Mobius differences was higher compared to the AcurosXB. Data input for the AcurosXB calculation model is more complex, and the algorithm was tuned using a variety of available information, including beam profiles, ionization‐chamber point measurements, gafchromic film measurements, and diode array measurements. In addition, AcurosXB is optimized for a large variety of clinical treatment scenarios. For these reasons, we are not surprised to see minor, more consistent, differences between AcurosXB calculations and point‐doses measured in the MVP.

Neither treatment technique, nor beam energy, had a significant effect on the differences seen between Eclipse AcurosXB and measurement. 6 MV VMAT plans showed an average difference of −1.4 ± 0.5%, while 10 MV VMAT plans showed an average difference of −1.7 ± 1.0%. 6 MV IMRT, 10 MV IMRT, and mixed‐beam IMRT show average differences of −1.1 ± 0.3%, −0.8 ± 0.3%, and −0.5 ± 0.4%, respectively. Similarly, Mobius did not show a significant difference in calculation accuracy due to treatment technique or beam energy. Six and 10 MV VMAT plans showed an average difference of 0.0 ± 1.5% and 0.5 ± 1.0%, respectively. 6 MV static IMRT showed an average difference of −1.3 ± 1.3%. 10 MV IMRT was evenly distributed, with an average difference of 0.1%, but a standard deviation of  ± 1.1%. Mixed‐beam IMRT plans showed an average difference of −1.0 ± 0.5%.

Overall, both the AcurosXB and Mobius dose calculation algorithms performed well compared to measurement. The more consistent results exhibited by the AcurosXB algorithm are expected due to the use of machine‐specific beam data, and a more robust internal MLC model. Numerous studies have been done examining the accuracy of the AcurosXB calculation algorithm and Eclipse MLC model, and point to its accuracy in a variety of clinical situations.^22^, ^23^, ^24^, ^25^ Despite the use of stock beam data, the Mobius dose calculation algorithm was capable of matching the measured dose within 3.5% for all measured cases.

### 3.B Pre‐clinical validation: Mobius vs. AcurosXB test case analysis

For each of the 17 previously delivered plans, TPS dose calculated with the AcurosXB algorithm was compared directly to dose calculated in Mobius on the patient CT‐dataset. The target mean dose percent difference, target D95% percent difference, 3D global gamma pass rate, and target gamma pass rate for each of the 17 analyzed plans can be found in Table 3. For reported percent differences, a positive percent difference indicates that Mobius calculated a higher number for a given statistic. For both the 3D global and target gamma pass rates, gamma criteria of 3%/2 mm were used. Mobius tended to report a higher target mean dose than AcurosXB, with an average target mean dose percent difference of 1.0 ± 1.1%. Target mean dose differences ranged from −1.2 to 2.6%. Mobius tended to report a lower target D95% than AcurosXB, with an average target D95% difference of −0.9 ± 2.0%. Target D95% percent differences ranged from −4.4 to 2.4%. 3D global gamma pass rates ranged from 87.9 to 99.9%, with an average value of 94.4 ± 3.3%. Gamma pass rate for the target‐only ranged from 58.5 to 98.6%, with an average value of 80.2 ± 12.3%.

**Table 3:** Comparison of patient dose calculated by Mobius to AcurosXB for pre‐clinical test cases

Plan number	Target mean dose percent difference (%)	Target D95% percent difference (%)	3D global gamma pass rate (%)	Target gamma pass rate (%)
1	1.4^a^	−0.4^a^	94.4^b^	79.4^b^
2	1.7	0.6	98.5	95.3
3	2.6	2.4	94.4	62.2
4	1.3	−1.3	99.9	98.0
5	1.5	−0.2	94.0	75.7
6	1.1	−0.5	95.2	83.0
7	1.8	0.1	95.7	69.4
8	1.8	−0.1	97.3	69.4
9	2.1	0.1	96.3	64.7
10	2.5	1.1	92.0	58.5
11	−0.6	−2.8	97.4	92.9
12	−1.2	−4.4	87.9	80.7
13	0.6	−3.7	89.9	82.1
14	−0.6	−3.2	88.7	87.7
15	0.2	−0.8	96.1	98.6
16	−0.4	−3.9	92.9	79.9
17	1.6	1.2	94.3	85.4

a A positive percent difference indicates that Mobius calculated dose is higher than AcurosXB.

b 3D global gamma pass rate and target gamma pass rate use gamma criteria of 3%, 2  mm.

**Figure 3:**
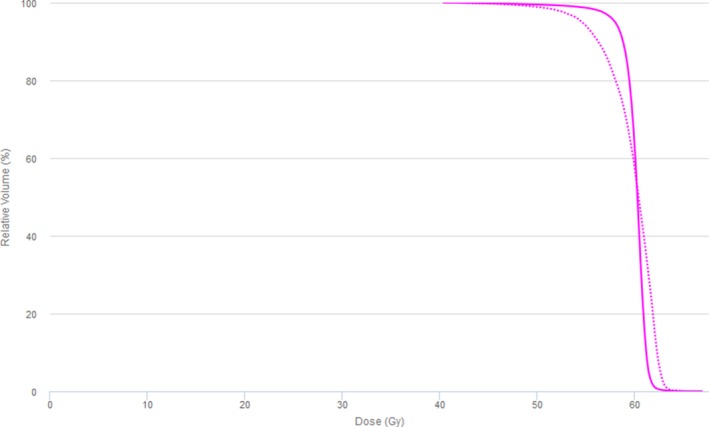
Target DVH calculated by Mobius and Eclipse AcurosXB. Typical target DVH curves calculated by Mobius (dotted) and Eclipse AcurosXB (solid) are shown.

**Figure 4:**
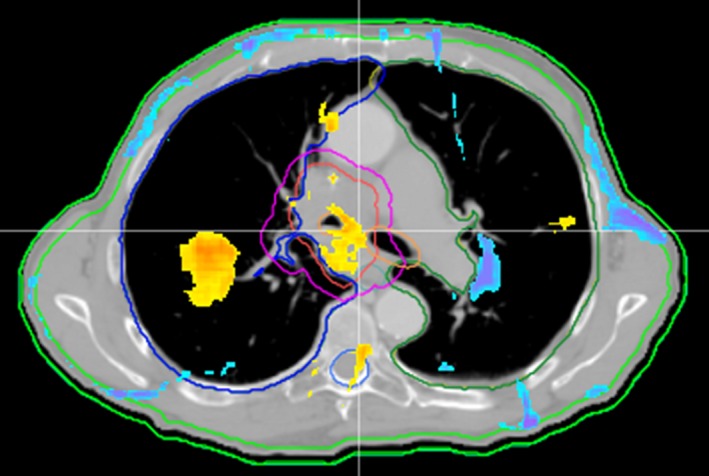
Mobius vs. Eclipse AcurosXB gamma map for selected treatment plan. Mobius vs. Eclipse AcurosXB gamma map (3%, 2 mm) is shown for treatment plan 16. Yellow and blue areas represent high and low gamma failures, respectively. Note the typical areas of gamma failure at each beam entrance due to differences in the build‐up model, and heterogeneous areas in the lung and bone.

Mobius tended to report a less homogeneous dose to the target compared to AcurosXB. Figure 3 shows a typical DVH comparison between AcurosXB and Mobius. Note the rounded shoulder and higher max dose of the Mobius calculation. This trend explains the tendency for Mobius to report a higher mean dose, and lower D95%. The dose differences reported by Mobius are likely due to dissimilarities between the two dose calculation algorithms and beam models. We found that surface dose was reported to be lower by Mobius than AcurosXB. Model variations in the dose build‐up region, a notoriously difficult area to model accurately, are likely responsible for this discrepancy. Material heterogeneity is also a likely source of dose difference between Mobius and AcurosXB. Mobius is a traditional collapsed cone convolution algorithm, which essentially calculates dose to non‐water materials utilizing density‐scaled water dose kernels.^1^ AcurosXB calculates dose to medium, requiring accurate assignment of materials within the body to incorporate the atomic properties of each material into the dose calculation.^26^ Because of the fundamentally different method each algorithm uses to account for heterogeneities, we expect to see dose differences in heterogeneous areas and at boundaries between differing materials. Figure 4, taken from plan 16, demonstrates each of these scenarios. Note the failing gamma points at each beam entrance due to differences in the build‐up model. In addition, multiple areas within the lung and bone also show failing gamma, likely due to differences in the way each algorithm handles inhomogeneities and re‐build‐up of dose. Further analysis is necessary to quantitate differences between the AcurosXB and Mobius dose algorithms due to patient anatomy.

### 3.C Post‐implementation: Mobius vs. AcurosXB clinical use analysis

Statistics for the first 36 clinical cases calculated with AcurosXB and Mobius can be found in Table 4. Average, maximum, minimum, and standard deviation values for the target mean dose percent difference, target D95% percent difference, 3D global gamma pass rate, target gamma pass rate, and point‐dose difference at a selected calculation point are listed. For reported dose differences, a positive percent difference indicates that Mobius calculated a higher number for a given statistic. For both the 3D global and target gamma pass rates, gamma criteria of 5%/2 mm were used.

**Table 4:** Mobius calculated patient dose vs. AcurosXB for first 36 intensity‐modulated cases^a^ post‐clinical implementation

	Target mean dose percent difference (%)	Target D95% percent difference (%)	3D global gamma pass rate (%)	Target gamma pass rate (%)	Dose calculation point percent difference (%)
Average	0.0^b^	−2.0^b^	99.2^c^	96.5^c^	1.8
Max	2.4	4.6	100.0	100.0	4.7
Min	−4.8	−10.0	96.7	69.0	−1.6
Standard deviation	1.6	2.4	0.8	5.6	1.3

a Analyzed cases include first 36 IMRT and VMAT plans treated post‐implementation of the Mobius system in our clinic.

b A positive percent difference indicates that the Mobius calculated dose is higher than AcurosXB.

c 3D global gamma and target gamma were calculated with a 5%, 2 mm criteria.

We found Mobius agreed well with AcurosXB over the 36 clinical intensity modulated treatment plans, representing a variety of treatment sites. The target mean dose percent difference, 3D global gamma pass rate, and point‐dose calculation were least sensitive to differences in the calculated dose distribution, with no clinical cases showing a difference greater than our clinical tolerances of 5% (dose difference) or 95% (gamma pass rate). Target D95%, was more sensitive, with three cases showing a difference greater than 5%. Gamma pass rate within the target‐only was most sensitive. Ten cases fell below 95% gamma pass rate, and two cases fell below 90% gamma pass rate. Plans showing differences outside our clinical tolerances were examined for the typical areas of disagreement outlined in the previous section. Of the two cases with target gamma pass rates below 90%, one involved a small PTV at the skin surface. The other case involved a small, low‐density lung PTV near a rib. Due to expected differences in the build‐up region, and at heterogeneous interfaces, the target gamma pass rate was affected significantly in each case. All differences were as expected, based on the pre‐clinical test cases.

## 4 Conclusion

The Mobius second check system was validated for a variety of intensity modulated clinical treatment plans, using the recently released MVP and an ADCL‐calibrated ionization chamber. We found that Mobius performed well compared to the AcurosXB dose calculation algorithm, implemented in our clinical treatment planning system, Eclipse. Mobius differences from measured dose were reasonable, especially in the context of a second check system utilizing a non‐customized beam model. We feel that intensity modulated plans can be safely verified using the Mobius system, and that extreme differences between Mobius and Eclipse would rightly prompt a thorough examination of the plan in question. The differences that are seen between Mobius and Eclipse are somewhat predictable, and are likely due to differences between the customized beam models in Eclipse, and the standard beam models in Mobius. Differences in the way AcurosXB handles heterogeneous materials and dose build‐up compared to Mobius also account for dissimilarities, especially for plans in anatomic areas with a high degree of heterogeneity. Notably, a recently released version of the Mobius software, v1.6, promises more accurate dose calculation in the build‐up region, which may improve agreement between Mobius and a primary TPS in this area.

## Conflict of Interest

The authors have no conflicts of interest to report.
